# Self-Rated Health as a Predictor of Disability Retirement – The Contribution of Ill-Health and Working Conditions

**DOI:** 10.1371/journal.pone.0025004

**Published:** 2011-09-20

**Authors:** Olli Pietiläinen, Mikko Laaksonen, Ossi Rahkonen, Eero Lahelma

**Affiliations:** Department of Public Health, Hjelt Institute, University of Helsinki, Helsinki, Finland; Erasmus University Rotterdam, The Netherlands

## Abstract

**Objective:**

Self-rated health is a generic health indicator predicting mortality, many diseases, and need for care. We examined self-rated health as a predictor of subsequent disability retirement, and ill-health and working conditions as potential explanations for the association.

**Methods:**

Self-rated health and the covariates were obtained from the Helsinki Health Study baseline mail surveys in 2000–2002 conducted among municipal employees aged 40–60 years (n = 6525). Data for disability retirement events (n = 625) along with diagnoses were linked from the Finnish Centre for Pensions, with a follow-up by the end of 2010. Hazard ratios (HR) and their 95% confidence intervals (CI) were calculated using competing risks models.

**Results:**

Less than good self-rated health predicted disability retirement due to all causes among both women (HR = 4.60, 95% CI = 3.84–5.51) and men (HR = 3.83, 95% CI = 2.64–5.56), as well as due to musculoskeletal diseases (HR = 5.17, 95% CI = 4.02–6.66) and mental disorders (HR = 4.80, 95% CI = 3.50–6.59) among women and men pooled. Ill-health and physical working conditions partly explained the found associations, which nevertheless remained after the adjustments. Among the measures of ill-health limiting long-standing illness explained the association most in all-cause disability retirement and disability retirements due to musculoskeletal diseases, whereas common mental disorders explained the association most in disability retirements due to mental health disorders. Among working conditions physical work load and hazardous exposures at work explained the association most, although much less than ill-health.

**Conclusions:**

Self-rated health is a strong predictor of disability retirement. This can be partly explained by ill-health and working conditions. Poor self-rated health provides a useful marker for increased risk of work disability and subsequent disability retirement.

## Introduction

Self-rated health is a commonly used generic health indicator. It is not directly concerned with any particular medical condition, but instead reflects broadly different domains of health that are not necessarily captured by specific measures of illness or disease [1]. Self-rated health has been found to reflect survey respondents' views of their health in terms of presence or absence of illnesses, functional limitations, and their subjective experience of health [2]. Possibly due to its inclusive and comprehensive nature self-rated health has predicted many health outcomes, such as functional limitations [3]–[5], use of health care services [6] and mortality [7]–[8] .

As poor self-rated health predicts many subsequent health outcomes, the question can be raised whether it also predicts disability retirement. Early retirement due to disability is a serious labour market issue in many countries aiming to help people continue their work career until old-age retirement. In Finland 7.5% of the working aged population received disability pension in 2009 [9]. The Finnish disability retirement scheme requires a diagnosed disease leading to long term inability to continue one's work, and the assessment of disability includes consideration of both health and work related characteristics [9]. In addition to being a labour market issue, disability retirement can also be used as an indicator of health and functioning based on a thorough assessment by medical professionals and requiring a severe functionally limiting disease.

We have identified four previous studies examining the association between self-rated health and subsequent disability retirement. In a cohort of middle-aged men from eastern Finland self-rated health predicted all-cause disability retirement after adjusting for baseline ill-health, socioeconomic position and health behaviours, with a hazard ratio of 2.7 for poor self-rated health [10]. In a cohort study on Swedish middle-aged men self-rated health predicted disability retirement with a relative risk of 3.7 for less than perfect self-rated health [11]. This association remained after adjusting for pre-existing diseases, albeit slightly reduced. Poor self-rated health also predicted disability retirement in two other studies examining a broad range of determinants of disability retirement [12]–[13]. Possible explanations for the association between self-rated health and disability retirement were not examined in these two studies.

Prior ill-health has partly explained the association of self-rated health and disability retirement [11]. However, previous studies have not systematically examined the two dimensions of work disability, namely ill-health and working conditions, as factors explaining the association between self-rated health and subsequent disability retirement. It is important to consider also working conditions as potential contributors to the association, since disability retirement first and foremost concerns the inability to continue one's work, and therefore neglecting the role of working conditions disregards an important part of the disability retirement process.

The aims of this study were to examine 1) to what extent self-rated health predicts disability retirement, and 2) whether ill-health and working conditions explain the association between self-rated health and disability retirement. We used survey data from the City of Helsinki employees with a prospective linkage to a national register on retirement. In addition to all retirements we separately examined disability retirement due to musculoskeletal diseases and mental disorders, the two most common disability retirement categories covering two thirds of all retirement events in Finland [9].

## Methods

### Ethics statement

The study has been approved by the Ethical Committee of the Department of Public Health, University of Helsinki, and the Ethical Committee of the City of Helsinki. Informed, written consent was obtained from all participants in the study.

### Data sources

The data for this study were derived from the Helsinki Health Study cohort on the staff of the City of Helsinki. The City of Helsinki is the largest employer in Finland with almost 40000 employees, and it provides basic services, including social and health care, education and cultural services, public transportation, environmental and technical maintenance as well as public administration. The staff includes hundreds of different blue-collar and white-collar occupations. The baseline survey was sent to the employees of the City of Helsinki, aged 40, 45, 50, 55, and 60 years, and conducted in 2000, 2001 and 2002 (n = 8960, response rate 67%, 80% of the respondents women).

The respondents to the survey who gave a permission for a register linkage (n = 6606) were followed up using the Finnish Centre for Pensions registers until the end of 2010. The register data contain information on all granted pensions during the follow-up period, including disability pensions with diagnoses. Information on deaths during the follow-up was obtained from the City of Helsinki and the Finnish Centre for Pensions registers. The respondents who had left employment at the City of Helsinki but had not received any retirement and therefore had no entries in the Finnish Centre for Pension registers had missing information on the possible time of death. Among women 508 (10%) of the respondents retired due to disability, 708 (14%) retired due to old age, 37 (1%) died and 855 (17%) became 63 years old, thereby becoming ineligible to enter disability retirement according to the current legislation (table 1). Among men 117 (8%) retired due to disability, 188 (13%) retired due to old age, 25 (2%) died and 335 (24%) became 63 years old during the follow-up. Of the 508 female disability retirees 127 (25%) retired due to mental disorders and 231 (45%) due to musculoskeletal disorders. Of the 117 male disability retirees 38 (32%) retired due to mental disorders and 38 (32%) due to musculoskeletal disorders.

**Table 1:** Distributions of variables.

		Women	Missing, n	Men	Missing, n
Retirement events	Disability retirement (n and %)				
	All diagnoses	508 (10)	0	117 (8)	0
	Mental disorders	127 (2)	0	38 (3)	0
	Musculosceletal diseases	231 (5)	0	38 (3)	0
	Other Diagnoses	150 (3)	0	41 (3)	0
	Retired for old age (n and %)	708 (14)	0	188 (13)	0
	Dead (n and %)	37 (1)	1081	25 (2)	318
	Become 63 years old (n and %)	855 (17)	0	335 (24)	0
Ill-health	Pre-existing diseases (mean and 95% CI)	2.36 (2.31, 2.42)	0	1.73 (1.65, 1.82)	0
	GHQ (mean and 95% CI)	1.92 (1.83, 2.00)	27	1.79 (1.63, 1.95)	10
	Limiting longstanding illnesses (mean and 95% CI)	0.17 (0.16, 0.18)	152	0.17 (0.15, 0.19)	37
	Sickness absence >2 weeks (mean and 95% CI)		0		0
	1 absence	0.20 (0.19, 0.21)		0.17 (0.15, 0.19)	
	2 or more absences	0.10 (0.09, 0.11)		0.06 (0.05, 0.07)	
	Purchased prescribed medication (mean and 95% CI)	0.20 (0.19, 0.21)	0	0.12 (0.10, 0.13)	0
	Has hospitalizations in the past 3 yrs (mean and 95% CI)	0.28 (0.26, 0.29)	0	0.22 (0.20, 0.24)	0
	Special reimbursement (mean and 95% CI)	0.29 (0.27, 0.30)	0	0.31 (0.28, 0.33)	0
Working conditions	Shift work (mean and 95% CI)	0.16 (0.15, 0.17)	37	0.22 (0.20, 0.24)	8
	Temporary work contract (mean and 95% CI)	0.12 (0.11, 0.13)	73	0.08 (0.07, 0.09)	15
	Working overtime (mean and 95% CI)	0.13 (0.12, 0.14)	71	0.21 (0.19, 0.24)	18
	Hazardous exposures (mean and 95% CI)	−0.06 (−0.09, −0.04)	312	0.13 (0.06, 0.19)	50
	Physical work load (mean and 95% CI)	0.06 (0.03, 0.09)	250	−0.43 (−0.47, −0.38)	43
	Computer work (mean and 95% CI)	−0.02 (−0.04, 0.01)	170	0.16 (0.12, 0.21)	16
	Low control (mean and 95% CI)	−0.05 (−0.08, −0.03)	151	0.06 (0.00, 0.12)	28
	High demans (mean and 95% CI)	0.00 (−0.03, 0.03)	313	−0.06 (−0.11, −0.01)	44
	Social support at work (mean sources of support, max 4)	0.70 (0.67, 0.73)	0	0.57 (0.52, 0.62)	0
Total (n)		5094		1395	

Furthermore, other national registers were used to derive measures of ill-health prior to the baseline survey used as explanatory factors in the analyses.

#### Self-rated health

Data on *self-rated health* was obtained from the baseline survey. Self-rated health was asked with a question “Generally speaking, how would you describe your health status: excellent, very good, good, fair, poor?” The measure was dichotomized to fair and poor indicating *less than good self-rated health* and the rest in another category.

#### Ill-health

We obtained information on ill-health from the baseline survey as well as various national registers. Three ill-health measures were obtained from the survey. *Self-reported lifetime diseases* diagnosed by a doctor were measured by calculating a sum of responses to 28 questions on individual diseases. *Limiting longstanding illness* was measured by two questions: whether the respondent has any longstanding illness, and whether it limits daily activities. These were combined to yield a measure of limiting longstanding illness. The General Health Questionnaire 12-item version (GHQ) was used to measure *common mental disorders* [14] .

Four indicators of ill-health were obtained from register sources. Data on *long-term sickness absence*, *prescribed medication purchases* and *eligibility for special reimbursement medication* were obtained from the registers of the Social Insurance Institution of Finland, and data on *hospitalizations* from the National Institute for Health and Welfare registers. *Long-term sickness absence* was measured by counting the number of sickness absences at least two weeks long during four to one years before the baseline, categorized to zero, one, or two or more absences. The absences one year prior to the baseline were not counted, as one year of sickness absence is typically required for disability retirement. *Prescribed medication purchases* were measured by counting these purchases during three years before baseline, dichotomized to those having over ten purchases and those having less. *Being eligible for special reimbursement* the respondent has to have a severe longstanding disease diagnosed by a doctor, for which medication is needed that is accepted for special reimbursement. *Hospitalizations* were dichotomized to no hospitalizations versus having had at least one hospitalization during three years before baseline.

#### Working conditions

Work arrangements as well as physical and psychosocial working conditions were used as measures of working conditions. Among work arrangements, data on *temporary work contract* at baseline was obtained from the City of Helsinki registers, and dichotomized to those with temporary work contract and those not. Other working conditions were obtained from the baseline survey. *Shift work* was dichotomized to those doing shift work and those not. *Working overtime* was dichotomized to those working over 40 hours per week and those working less.

Physical working conditions were measured by eighteen questions regarding presence and severity of various working environment factors. They were summarized to three measures on the basis of factor analysis: *hazardous exposures at work* consisting of nine questions, *physical work load* consisting of six questions and *computer work* consisting of three questions. The responses on each factor were added together and standardized to have a mean of zero and a standard deviation of one among women and men together. Psychosocial working conditions were measured by nine questions on *job demands* and nine questions on *job control*, following the Framingham version of Karasek's job-demand-control inventory [15]. The responses among both measures of psychosocial working conditions were added together and standardized to have a mean of zero and a standard deviation of one among women and men together. *Social support at work* was measured by Sarason's brief inventory [16] counting the answers to the four questions on support received from co-workers or the supervisor when in need of help.

### Statistical methods

Competing risks models were used to calculate hazard ratios and their 95% confidence intervals. These models are intended for situations with multiple possible outcomes, where the study participant is followed up until the first outcome [17]. Competing risks models were preferred to Cox regression since the study participants face the possibility of not only disability retirement, but also old age retirement and death. In addition the study participants might also face another event, reaching the age of 63 years, thereby becoming ineligible for disability retirement. Because this event is reached by every participant of sufficient age, it was not included as a competing risk in the models, but the participants were censored when reaching that age. In the analyses of disability retirement events due to musculoskeletal diseases and mental disorders, both of these diagnoses as well as a separate group of all other diagnoses were included as competing outcomes in the same model. Age at the baseline and age at the first event were used as the dependent variable in the models, thereby rendering separate age adjustment redundant.

To assess the contribution of baseline ill-health and working conditions to the association between self-rated health and disability retirement, measures of ill-health and working conditions were added to the base model one by one, as well as groups of all ill-health measures together, all working conditions together and all explanatory factors together.

Multiple imputation for missing values on the explanatory factors was conducted using the aregImpute function in the Hmisc package [18] for R software [19]. This function uses additive regression, bootstrapping, and predictive mean matching for the imputation. The imputation process was used to create ten imputed datasets, and the data were assumed missing at random.

## Results

We first examined to what extent self-rated health at baseline predicted subsequent all-cause disability retirement, and the contribution of ill-health and working conditions to this association. For women with less than good self-rated health the hazard ratio for disability retirement was 4.60 (95% CI 3.84 to 5.51) (table 2). Adjusting for limiting longstanding illness, sickness absences, pre-existing diseases, prescribed medication purchases, eligibility for special reimbursement and GHQ score decreased the hazard ratio. The health indicator explaining the association most was limiting longstanding illness, which decreased the hazard ratio to 2.97 (95% CI 2.40 to 3.67). Together all health indicators explained over 50 percent of the association of self-rated health and disability retirement. Adjusting for physical work load reduced the hazard ratio to 3.80 (95% CI 3.13 to 4.61), while other working conditions had negligible effects. Together all working conditions explained around 20 percent of the association between self-rated health and disability retirement among women, and adjusting for working conditions in addition to ill-health did not have much effect. After all adjustments the hazard ratio for less than good self-rated health was 1.86 (95% 1.43 to 2.42).

**Table 2:** Hazard ratios and 95% CI for less than good self-rated health for all cause disability retirement with explanatory factors adjusted for, women and men separately and together.

		All diagnoses					
		Women		Men		Women and men	
		Hazard ratio	% explained	Hazard ratio	% explained	Hazard ratio	% explained
**M1**	**Base model**	**4.60 (3.84, 5.51)**		**3.83 (2.64, 5.56)**		**4.48 (3.81, 5.27)**	
M2	M1 + Pre-existing diseases	3.56 (2.94, 4.31)	23	2.99 (2.01, 4.46)	22	3.48 (2.93, 4.13)	22
M3	M1 + GHQ	4.00 (3.40, 4.85)	13	2.92 (1.95, 4.38)	24	3.77 (3.17, 4.49)	16
M4	M1 + Limiting longstanding illnesses	2.97 (2.40, 3.67)	35	2.79 (1.85, 4.21)	27	3.02 (2.50, 3.63)	33
M5	M1 + Sickness absence > 2 weeks	3.53 (2.92, 4.26)	23	3.10 (2.11, 4.56)	19	3.47 (2.93, 4.11)	23
M6	M1 + Prescribed medication purchases	3.98 (3.30, 4.79)	14	3.69 (2.52, 5.39)	4	3.94 (3.34, 4.65)	12
M7	M1 + Hospitalizations	4.23 (3.52, 5.08)	8	3.34 (2.29, 4.88)	13	4.08 (3.46, 4.81)	9
M8	M1 + Special reimbursement	3.95 (3.29, 4.75)	14	3.24 (2.21, 4.76)	15	3.84 (3.26, 4.54)	14
**M9**	**M1 + All ill-health measures**	**2.00 (1.60, 2.51)**	**57**	**1.67 (1.06, 2.62)**	**56**	**1.98 (1.62, 2.42)**	**56**
M10	M1 + Shift work	4.57 (3.81, 5.48)	1	3.77 (2.59, 5.49)	2	4.44 (3.77, 5.22)	1
M11	M1 + Temporary work contract	4.71 (3.92, 5.65)	−3	3.87 (2.65, 5.63)	−1	4.56 (3.87, 5.37)	−2
M12	M1 + Working overtime	4.51 (3.76, 5.41)	2	3.75 (2.56, 5.48)	2	4.39 (3.73, 5.17)	2
M13	M1 + Hazardous exposures at work	4.24 (3.50, 5.14)	8	3.27 (2.21, 4.84)	15	4.07 (3.42, 4.83)	9
M14	M1 + Physical work load	3.80 (3.13, 4.61)	17	3.45 (2.34, 5.09)	10	3.78 (3.18, 4.49)	16
M15	M1 + Computer work	4.53 (3.76, 5.46)	2	3.83 (2.63, 5.57)	0	4.47 (3.79, 5.28)	0
M16	M1 + Low control at work	4.20 (3.48, 5.06)	9	3.48 (2.36, 5.14)	9	4.08 (3.45, 4.83)	9
M17	M1 + High demands at work	4.54 (3.75, 5.48)	1	3.78 (2.58, 5.54)	1	4.44 (3.75, 5.26)	1
M18	M1 + Social support at work	4.54 (3.78, 5.45)	1	3.75 (2.57, 5.46)	2	4.42 (3.75, 5.20)	1
**M19**	**M1 + All working conditions**	**3.64 (2.93, 4.52)**	**21**	**2.96 (1.90, 4.61)**	**23**	**3.56 (2.93, 4.32)**	**21**
**M20**	**M1 + All predictors**	**1.86 (1.43, 2.42)**	**60**	**1.36 (0.80, 2.31)**	**64**	**1.83 (1.45, 2.31)**	**59**

For men with less than good self-rated health the hazard ratio for all cause disability retirement was 3.83 (95% CI 2.64 to 5.56). Adjusting for measures of ill-health other than prescribed medication purchases weakened the association of self-rated health and disability retirement. The factor explaining the association the most was limiting longstanding illness, which decreased the hazard ratio to 2.79 (95% CI 1.85 to 4.21). Adjusting for all measures of ill-health explained over half of the association of self-rated health and disability retirement among men. Adjusting for hazardous exposures at work and physical work load also reduced the hazard ratio. Hazardous exposures explained the association most, reducing the hazard ratio to 3.27 (95% CI 2.21 to 4.84). Together all working conditions explained about 20 percent of the association between self-rated health and disability retirement among men, and adjusting for both working conditions and ill-health explained over 60 percent of the association. After all adjustments the association between self-rated health and disability retirement was not statistically significant among men.

Among women and men pooled together self-rated health predicted disability retirement strongly. Adjusting for all measures of ill-health excluding hospitalizations, as well as physical work load partly explained the association of self-rated health and disability retirement, and together all explanatory factors explained around 60 percent of the association, which nevertheless remained nearly twofold after all adjustments.

Next we examined to what extent less than good self-rated health predicted disability retirement due to mental disorders, musculoskeletal disorders and all other diseases combined (table 3). Women and men were pooled in these analyses due to low number of retirement events when stratified by gender. The hazard ratio of less than good self-rated health was 4.80 (95% CI 3.50 to 6.59) for disability retirement due to mental disorders in the gender adjusted base model. The unadjusted model showed similar results. Adjusting for common mental disorders, pre-existing diseases, limiting longstanding illness, sickness absence, prescribed medication purchases or eligibility for special reimbursement all reduced the hazard ratio somewhat. Together all measures of ill-health explained about 70 percent of the association of self-rated health and disability retirement due to mental disorders. Adjusting for any of the working conditions separately did not have much effect, but adjusting for all of them together explained about 20 percent of the association. Together all explanatory factors explained around 70 percent of the association, which was no longer statistically significant (HR 1.31, 95% CI 0.82–2.07).

**Table 3:** Hazard ratios and 95% CI for less than good self-rated health for disability retirement due to mental disorders, musculoskeletal diseases and other diagnoses with explanatory factors adjusted for, women and men together.

		Disability retirement due to mental disorders		Disability retirement due to musculoskeletal diseases		Disability retirement due to other diseases	
		Hazard ratio	% explained	Hazard ratio	% explained	Hazard ratio	% explained
**M0**	**Unadjusted model**	**4.79 (3.49, 6.57)**		**5.17 (4.02, 6.65)**		**3.49 (2.62, 4.66)**	
**M1**	**Base model (adjusted for gender)**	**4.80 (3.50, 6.59)**	**0**	**5.17 (4.02, 6.66)**	**0**	**3.50 (2.62, 4.67)**	**0**
M2	M1 + Pre-existing diseases	3.21 (2.29, 4.48)	33	4.20 (3.22, 5.47)	19	2.96 (2.18, 4.01)	15
M3	M1 + GHQ	2.88 (2.04, 4.07)	40	5.11 (3.92, 6.66)	1	3.15 (2.31, 4.29)	10
M4	M1 + Limiting longstanding illnesses	3.57 (2.47, 5.15)	26	3.29 (2.46, 4.40)	36	2.37 (1.71, 3.29)	32
M5	M1 + Sickness absence > 2 weeks	3.81 (2.75, 5.30)	21	3.89 (2.99, 5.06)	25	2.79 (2.07, 3.77)	20
M6	M1 + Prescribed medication purchases	4.03 (2.91, 5.59)	16	4.61 (3.56, 5.96)	11	3.16 (2.35, 4.25)	10
M7	M1 + Hospitalizations	4.63 (3.36, 6.38)	4	4.62 (3.58, 5.97)	11	3.12 (2.33, 4.18)	11
M8	M1 + Special reimbursement	4.11 (2.97, 5.68)	14	4.94 (3.82, 6.39)	4	2.57 (1.92, 3.46)	27
**M9**	**M1 + All ill-health measures**	**1.55 (1.04, 2.31)**	**68**	**2.73 (2.00, 3.72)**	**47**	**1.55 (1.09, 2.21)**	**56**
M10	M1 + Shift work	4.88 (3.54, 6.73)	−2	5.03 (3.90, 6.47)	3	3.47 (2.60, 4.65)	1
M11	M1 + Temporary work contract	4.83 (3.51, 6.64)	−1	5.39 (4.17, 6.97)	−4	3.51 (2.62, 4.68)	0
M12	M1 + Working overtime	4.68 (3.40, 6.45)	3	5.17 (4.01, 6.66)	0	3.34 (2.49, 4.48)	5
M13	M1 + Hazardous exposures at work	4.49 (3.22, 6.27)	6	4.52 (3.46, 5.91)	13	3.27 (2.39, 4.46)	7
M14	M1 + Physical work load	4.78 (3.44, 6.65)	0	3.71 (2.82, 4.88)	28	3.13 (2.31, 4.24)	11
M15	M1 + Computer work	4.59 (3.32, 6.36)	4	5.30 (4.09, 6.85)	−3	3.43 (2.55, 4.61)	2
M16	M1 + Low control at work	4.46 (3.20, 6.21)	7	4.58 (3.53, 5.95)	11	3.27 (2.43, 4.41)	7
M17	M1 + High demans at work	4.79 (3.43, 6.69)	0	5.25 (4.03, 6.84)	−2	3.37 (2.50, 4.54)	4
M18	M1 + Social support at work	4.72 (3.43, 6.49)	2	4.98 (3.86, 6.42)	4	3.58 (2.67, 4.78)	−2
**M19**	**M1 + All working conditions**	**3.84 (2.65, 5.57)**	**20**	**3.55 (2.61, 4.84)**	**31**	**3.28 (2.33, 4.62)**	**6**
**M20**	**M1 + All predictors**	**1.31 (0.82, 2.07)**	**73**	**2.26 (1.56, 3.27)**	**56**	**1.65 (1.10, 2.47)**	**53**

Less than good self-rated health predicted also disability retirement due to musculoskeletal diseases, with a hazard ratio of 5.17 (95% CI 4.02 to 6.66) in the gender adjusted base model. Adjusting for limiting longstanding illnesses, sickness absence, pre-existing diseases, prescribed medication purchases and hospitalizations reduced the hazard ratio somewhat. Together all measures of ill-health explained around half of the association between self-rated health and disability retirement. Adjusting for physical work load, hazardous exposures at work and low control at work all somewhat reduced the hazard ratio. Together the working conditions explained approximately one third of the association, and all predictors together explained over 50 percent. The association remained after adjustments.

Self-rated health also predicted disability retirement due to other causes, although the association was weaker than in the two most common diagnosis groups. Largely the same factors explained the association as with musculoskeletal diseases, but eligibility for special reimbursement was clearly a stronger predictor for disability retirements due to other causes. Working conditions did not notably explain the association between self-rated health and retirement due to other diseases, which remained statistically significant after all adjustments.

## Discussion

Our analyses showed that self-rated health strongly predicts disability retirement among both women and men over a follow-up of on average 9 years. This holds for disability retirement due to all causes, as well as due to mental health disorders and musculoskeletal diseases, and any other diseases combined. These results are in accordance with previous studies on the association of self-rated health with subsequent disability retirement [10]–[13].

Ill-health explained around half of the association of self-rated health with subsequent disability retirement due to all causes and musculoskeletal diseases, and almost 70 percent of the association with disability retirement due to mental disorders. For all-cause disability retirement and disability retirement due to musculoskeletal diseases limiting longstanding illness explained this association most, whereas for disability retirement due to mental disorders the strongest explanatory factor was common mental disorders, as measured with the GHQ. Strong associations with these health measures may be partly explained by their generic nature. Limiting longstanding illness is a generic measure of health which is correlated with self-rated health, and it reflects functional limitations due to ill-health, which is a necessary condition for disability retirement. GHQ measures mental health, which is by definition closely related to disability retirement due to mental disorders. In a previous study on Swedish middle-aged men prior ill-health also explained the association of self-rated health with subsequent disability retirement, but the association remained [11].

Working conditions explained around 20 percent of the association of self-rated health with subsequent disability retirement due to all causes and due to mental disorders, and slightly more of the association with disability retirement due to musculoskeletal diseases. The last mentioned are mainly explained by physical work load.

Our analyses showed that ill-health explained more of the association between self-rated health and disability retirement than working conditions. However, the relative significance of working conditions compared to ill-health was larger for disability retirement due to musculoskeletal diseases than for all cause disability retirement or disability retirement due to mental disorders. Thus ill-health was clearly a stronger predictor of disability retirement due to mental disorders, than of that due to musculoskeletal diseases. GHQ explained a large part of the association for disability retirement due to mental disorders but was unimportant for retirements due to musculoskeletal diseases. The association of self-rated health with subsequent disability retirement remained after adjusting for ill-health and working conditions in all-cause disability retirements and disability-retirements due to musculoskeletal diseases, but not in disability retirements due to mental disorders . Poor self-rated health was a stronger predictor for disability retirement among women, but largely the same factors explained the association among both women and men.

Why does self-rated health predict disability retirement even independently of ill-health and working conditions? Working conditions do not exhaust the association of self-rated health and disability retirement possibly because ill-health is the primary and necessary condition when the assessment on granting disability retirement is made, while working conditions are considered only secondarily. Therefore it is understandable that working conditions explain the association of self-rated health with disability retirement to a lesser degree.

The reasons for the association of self-rated health and disability retirement remaining after adjusting for ill-health cannot be directly judged from our analyses or previous research. However, we can apply here some of the explanations found in studies that have examined self-rated health as a predictor of further health related outcomes. It is unlikely that self-rated health is a causal predictor of disability retirement, but it is instead likely a thorough summary of the respondent's overall health. It is possible that self-rated health captures domains of health that are not covered by other health indicators because of practical limitations in empirical studies such as inadequate measurement , or our currently limited understanding of how to measure them [1]. Individuals can take into account all aspects of health they see relevant, such as familial risk factors or severity and prognosis of the disease, whereas in population studies it is not practically possible to measure all relevant information on health. Self-rated health may also reflect health dynamically in time, including declining health, whereas most other health measures reflect health in a more static way [8].

Self-rated health has previously been studied as a predictor of health-related outcomes, but the majority of these studies have aimed to predict mortality. Mortality has been called the strongest biological indicator of ill-health [1]. However, mortality does not capture the full range of health, but reflects primarily fatal diseases, disregarding functional limitations and other lesser health problems that do not lead to death. There are some studies that have found self-rated health to be a predictor of less severe health outcomes, such as functional limitations [3]–[4] and health care utilization [6]. In our study self-rated health predicted mental disorders and musculoskeletal diseases, both diagnosis groups usually not fatal, more than other diagnosis groups, which also include fatal diseases. Because disability retirement serves as a measure of reduced functioning, our study also contributes to the research on self-rated health as a predictor of functional limitations.

### Methodological considerations

Certain characteristics of our study give credibility to our results. Our study combines survey data from a reasonably large cohort of employees with a set of both register based and self-reported health measures more comprehensive than those assessed in previous studies. As argued by Jylhä [1], it is valuable to include both self-reported and more objective health measures in studies on self-rated health, because if all health measures in the analysis are based on self-report they are likely to be modified by the same evaluation framework used by the respondent. To our knowledge our study is also the first to assess both ill-health and working conditions as possible explanations for the association of self-rated health and disability retirement. With the exception of one study [12] previous studies on the association of self-rated health with subsequent disability retirement have included only men. Our study includes both women and men, and it is the first to examine explanatory factors of the association among women.

Our study has also some limitations. An examination by a physician would provide even more comprehensive evaluation of ill-health, although the register-based measures of ill-health are based on a diagnosis from a doctor and the survey questions on pre-existing diseases specifically ask of diseases diagnosed by a physician. Non-response reduced the original survey sample by 33%, and declining linkage to external registers further to 50% of the original, which may cause bias to the results. However, non-response analysis made on the baseline survey suggests non-response and declining the linkage to be unlikely to cause bias to the results based on the data [20]. Multiple imputation has been used to account for missing data on individual variables, but it cannot account for completely missing cases. Our results are based on a cohort of employees, and therefore the sample is not fully representative of the whole population of Finland. Nevertheless, City of Helsinki is the largest employer in Finland, and our results can be generalized with caution to the municipal workforce.

### Conclusions

Self-rated health is a strong predictor of all-cause disability retirement as well as disability retirement due to mental disorders and musculoskeletal diseases. The association can be partly explained by ill-health and working conditions, but self-rated health is likely to have predictive power independent of these. In our study prior-ill health was well covered by indicators based on both questionnaire and register-based data sources. Poor self-rated health provides a useful marker for increased risk of work disability and subsequent disability retirement.

## References

[1] Jylhä M (2009). What is self-rated health and why does it predict mortality? Towards a unified conceptual model.. Soc Sci Med.

[2] Manderbacka K (1998). Examining what self-rated health question is understood to mean by respondents.. Scand J Soc Med.

[3] Lee Y (2000). The predictive value of self assessed general, physical, and mental health on functional decline and mortality in older adults.. Journal of Epidemiology and Community Health.

[4] Lee Y, Shinkai S (2003). A comparison of correlates of self-rated health and functional disability of older persons in the Far East: Japan and Korea.. Arch Gerontol Geriatr.

[5] Idler EL, Russell LB, Davis D (2000). Survival, functional limitations, and self-rated health in the NHANES I Epidemiologic Follow-up Study, 1992. First National Health and Nutrition Examination Survey.. Am J Epidemiol.

[6] Miilunpalo S, Vuori I, Oja P, Pasanen M, Urponen H (1997). Self-rated health status as a health measure: the predictive value of self-reported health status on the use of physician services and on mortality in the working-age population.. J Clin Epidemiol.

[7] DeSalvo KB, Bloser N, Reynolds K, He J, Muntner P (2006). Mortality prediction with a single general self-rated health question. A meta-analysis.. J Gen Intern Med.

[8] Idler EL, Benyamini Y (1997). Self-rated health and mortality: a review of twenty-seven community studies.. J Health Soc Behav.

[9] Tilasto Suomen eläkkeensaajista 2009 (n.d.).. http://www.etk.fi/Binary.aspx?Section=42845&Item=64965.

[10] Karpansalo M, Manninen P, Kauhanen J, Lakka TA, Salonen JT (2004). Perceived health as a predictor of early retirement.. Scand J Work Environ Health.

[11] Månsson NO, Råstam L (2001). Self-rated health as a predictor of disability pension and death–a prospective study of middle-aged men.. Scand J Public Health.

[12] Krokstad S, Johnsen R, Westin S (2002). Social determinants of disability pension: a 10-year follow-up of 62 000 people in a Norwegian county population.. International Journal of Epidemiology.

[13] Biering-Sørensen F, Lund J, Høydalsmo OJ, Darre EM, Deis A (1999). Risk indicators of disability pension. A 15 year follow-up study.. Dan Med Bull.

[14] Goldberg D (1972). The Detection of Psychiatric Illness by Questionnaire.

[15] Karasek RA (1979). Job Demands, Job Decision Latitude, and Mental Strain: Implications for Job Redesign.. Administrative Science Quarterly.

[16] Sarason IG (1983). Assessing social support: The social support questionnaire.. J Pers Soc Psychol.

[17] Putter H, Fiocco M, Geskus RB (2007). Tutorial in biostatistics: competing risks and multi-state models.. Statist Med.

[18] Alzola C, Harrell F (2006). An Introduction to S and the Hmisc and DesignLibraries.. http://biostat.mc.vanderbilt.edu/twiki/pub/Main/RS/sintro.pdf.

[19] R Development Core Team (2011). R: A Language and Environment for Statistical Computing..

[20] Laaksonen M, Aittomäki A, Lallukka T, Rahkonen O, Saastamoinen P (2008). Register-based study among employees showed small nonparticipation bias in health surveys and check-ups.. J Clin Epidemiol.

